# Chromosome 17q12 deletion, including *HNF1B* presenting with recurrent severe cholestasis and profound hypomagnesemia during pregnancy: a case report

**DOI:** 10.1016/j.xagr.2026.100635

**Published:** 2026-03-25

**Authors:** Nina Boe, Herman Hedriana, Krishna Singh

**Affiliations:** 1 Department of Obstetrics and Gynecology, UC Davis Health, Sacramento, CA (Boe, Hedriana, Singh); 2 Division of Maternal-Fetal Medicine, UC Davis Health, Sacramento, CA (Boe, Hedriana, Singh)

**Keywords:** 17q12 deletion, case report, cholestasis, pregnancy

## Abstract

**Introduction:**

17q12 deletion features kidney/genital abnormalities, maturity-onset diabetes of the young, and bile duct hypoplasia. The condition may first present with severe cholestasis during pregnancy, and is important to differentiate from classic intrahepatic cholestasis of pregnancy.

**Case Presentation:**

A 30-year-old G3P0202 was referred at 27 weeks’ gestation with generalized weakness exacerbated in pregnancy. Obstetric history was significant for two prior spontaneous preterm births in the setting of a bicornuate uterus. The patient had severe cholestasis during both prior pregnancies. Family history is significant for a son with severe hydronephrosis and autism. The patient’s laboratories revealed critically low serum magnesium with severely elevated 24-hour-urine magnesium, suggesting renal magnesium wasting. She was treated with ongoing magnesium infusions. She was also diagnosed with severe cholestasis and gestational diabetes, and ultimately induced at 33 weeks due to intractable pruritus. The infant had a 35-day stay in the neonatal intensive care unit. Maternal chromosomal microarray returned with a pathogenic deletion of 17q12. The patient was advised to have her children evaluated with a geneticist.

**Conclusion:**

17q12 deletion was the likely etiology of recurrent severe cholestasis complicating our patient’s prior pregnancies, and in the current pregnancy, along with renal dysfunction. Knowledge of 17q12 deletion is important for counseling of affected reproductive-aged women at risk of stillbirth associated with severe cholestasis and renal dysfunction during pregnancy. 17q12 deletion should be considered in the differential diagnosis when severely elevated bile acids are encountered during pregnancy, particularly in combination with maternal Mullerian anomaly or renal dysfunction, or fetal renal anomaly.

**AJOG Global Reports at a Glance**

**Why was this study conducted?**

This study aims to increase awareness of 17q12 deletion syndrome as a potential etiology for severe cholestasis during pregnancy.

**Key findings**

Combination of severe cholestasis, tubulointerstitial renal disease, and urogenital anomaly complicating pregnancy are manifestations of 17q12 deletion syndrome.

**What does this add to what is known?**

17q12 deletion syndrome may first present with recurrent severe cholestasis and renal dysfunction in the form of profound hypomagnesemia during pregnancy. Identification of this syndrome allows for more comprehensive management and counseling regarding associated reproductive risks.

## Introduction

Deletion of 17q12 including *HNF1B* results in an autosomal dominant multi-system disorder with a complex phenotype: variable structural or functional abnormalities of the kidney and urogenital tract; neurodevelopmental or neuropsychiatric disorders including autism, seizures, anxiety, bipolar disorder, and schizophrenia; maturity-onset diabetes of the young (MODY) type 5; pancreatic hypoplasia with exocrine insufficiency and pancreatitis; poorly developed bile ducts with reduced bile flow; dysmorphic features; vision problems; structural brain anomalies; and sometimes cardiac defects (Table 1).^1^ We report on a patient with 17q12 deletion syndrome presenting with recurrent severe cholestasis and profound hypomagnesemia during pregnancy.

**Table 1:** 17q12 deletion syndrome: frequency of select features

Frequency	Features
Most common (>50%)	• Kidney structural or functional defects • Neurodevelopmental/neuropsychiatric disorders • Mild dysmorphic features
Common (25%–50%)	• Maturity-onset diabetes of the young type 5 • Genital abnormalities • Structural and functional liver abnormalities • Structural and exocrine abnormalities of the pancreas • Hyperparathyroidism • Eye abnormalities • Nonspecific structural brain findings
Less common (<25%)	• Congenital cardiac anomalies • Seizures

## Patient information

A 30-year-old G3P0202 was referred to our center in the third trimester with profound hypomagnesemia and cholestasis during pregnancy. She was initially seen at 27+3 weeks’ gestation at an outside hospital with vague abdominal pain. Laboratory evaluation identified critically low serum magnesium level of 0.5 mg/dL, mild hypokalemia (3.1 mmol/L), and mildly elevated liver enzymes (AST 103 U/L and ALT 165 U/L). Pre-eclampsia was considered but was thought unlikely since the patient was normotensive and urine protein was negative. Abdominal ultrasound showed mild liver enlargement and adenomyomatosis of the gallbladder. Viral and immune hepatitis testing returned negative. Bile acids were mildly elevated at 22 µmol/L (upper limit of normal 10 µmol/L). The patient received 10 grams of intravenous magnesium during the first day of hospital admission; however, levels remained below normal range. She was seen by a nephrologist and scheduled for magnesium infusions three times a week as an outpatient and was started on ursodiol for cholestasis.

The patient’s pregnancy history was significant for two prior spontaneous late preterm births presenting with preterm labor. Bicornuate uterus was identified on ultrasound during the first pregnancy. She had a history of gestational diabetes in the first pregnancy and cholestasis during both of her prior pregnancies (severe in the second), but was not known to have hypomagnesemia. She reported a long history of fatigue with generalized weakness and migraines even outside of pregnancy. Evaluations at that time revealed mild to moderate hypokalemia responsive to replacement. A CT of the brain was normal. She also has a history of generalized anxiety. She denied pruritus outside of pregnancy. Her family history is significant for her son, age 4, who had severe unilateral hydronephrosis requiring surgical correction, developmental delay, and autism.

## Clinical findings

On examination, the patient had minor dysmorphism, including frontal bossing, highly arched palate, lax joints, and fifth digit clinodactyly. Prenatal ultrasound in the current pregnancy was unremarkable with normal appearing fetal kidneys. Maternal renal ultrasound showed a one-to-two-centimeter simple cyst on each kidney.

## Timeline

**Table:** 

	27 wk	28 wk	30 wk	33 wk
**Magnesium**	0.5 mg/dL	2.1 mg/dL	1.2 mg/dL	0.8 mg/dL
**Potassium**	3.1 mmol/L	3.5 mmol/L	3.3 mmol/L	3.7 mmol/L
**Calcium**	8.6 mg/dL	8.9 mg/dL	8.9 mg/dL	10.8 mg/dL
**AST**	103 U/L	178 U/L	135 U/L	161 U/L
**ALT**	165 U/L	272 U/L	244 U/L	191 U/L
**Bile acids**	22 µmol/L	90 µmol/L	85 µmol/L	157 µmol/L
**Creatinine**	0.4 mg/dL	0.4 mg/dL	0.56 mg/dL	0.56 mg/dL
**24-h urine mg**		1039 mg/d (upper limit of normal 199)		
**Glucose screen**		221 mg/dL		
**HbA1c**	5.3%		5.6%	

## Diagnostic assessment

At 28+3 weeks, the patient returned with complaints of fatigue and weakness despite outpatient magnesium infusions, and she also reported increasing pruritus. She was transferred to our center for further evaluation.

No etiology to explain hypomagnesemia, such as precipitating medications, toxic exposures, recent episodes of vomiting or diarrhea, or other illnesses, was identified. The patient had normal serum creatinine, no evidence of acidosis, and normal parathyroid function. Twenty-four-hour urine magnesium was significantly elevated at 1039 mg/d (upper limit of normal 199 mg/d), suggesting primary renal magnesium wasting. Liver transaminases remained elevated, and bile acids had increased to 90 µmol/L. Gestational diabetes testing returned abnormal with 1-hour blood glucose of 221 mg/dL. The patient was started on a diabetic diet. A PICC line was placed to facilitate intravenous magnesium repletion, which was complicated by SVT, and treated with a beta blocker. Her echocardiogram was normal. The patient required ongoing magnesium repletion and was started on a potassium-sparing diuretic by our nephrologist, who suspected *HNF1B-*related renal cysts and diabetes syndrome. Medical genetics was consulted for further evaluation.

Maternal chromosomal microarray analysis was ordered, along with a 38-gene panel for hypokalemia related disorders at GeneDx Laboratories, which included the *HNF1B* gene. In addition, a six-gene panel for familial intrahepatic cholestasis was also sent to GeneDx Laboratories, which included the genes *ABCB11, ABCB4, ATP8B1, JAG1, NOTCH2,* and *TJP2*. The chromosomal microarray revealed a 1377 kb pathogenic deletion of 17q12 (34815072_36192489) x1, and the hypokalemia panel returned with a heterozygous pathogenic deletion of the *HNF1B* gene. The familial cholestasis panel was negative.

## Therapeutic interventions

The patient was discharged at 29+2 weeks but had two additional admissions for persistent fatigue and refractory hypomagnesemia despite outpatient magnesium infusions. She was ultimately scheduled for labor induction at 33 weeks due to severely elevated, rapidly rising bile acids, peaking at 157 µmol/L, with intractable pruritus.

## Follow-up and outcomes

The patient had an uncomplicated vaginal delivery. The infant was transferred to the neonatal intensive care unit with respiratory distress and was discharged in stable condition on day of life 35. The patient was scheduled for follow-up with nephrology, endocrinology, gastroenterology, and ophthalmology, and she was advised to have her children evaluated by pediatric genetics for the known maternal chromosomal deletion.

## Discussion

17q12 deletion syndrome is usually diagnosed when a patient presents with diabetes that becomes recognized as MODY, or when cholestasis is encountered outside of pregnancy. Hypomagnesemia concurrent with diabetes or cholestasis is also a characteristic finding. For patients with known 17q12 deletion syndrome, recommended surveillance includes: monitoring of blood pressure, assessments of kidney function (serum magnesium and potassium concentrations and protein-to-creatine ratios), renal ultrasounds every 3 to 5 years, psychoeducational evaluations, evaluation for uterine and vaginal abnormalities related to Mullerian duct aplasia, annual hemoglobin A1c levels, periodic hepatic and lipid panels, assessment for hyperparathyroidism, and ophthalmologic and hearing screening.^1^ Preconception consultation is important for affected reproductive-aged women, particularly for review of potential obstetric complications which may include pre-eclampsia (associated with diabetic nephropathy and/or *HNF1B*-related tubulointerstitial disease), cholestasis, preterm delivery, diabetes, worsening renal disease, and 50% risk of inheritance of the condition among offspring.

There is paucity of information regarding pregnancy outcomes among women with known 17q12 deletion. Four cases identified in the literature revealed one woman with diabetes, hypomagnesemia, and renal cysts who developed pre-eclampsia in her first pregnancy and refractory hypomagnesemia during her second pregnancy, but both delivered at term; another with cholestasis and hypomagnesemia outside of pregnancy who was identified with large kidneys on fetal ultrasound and developed diabetes during pregnancy^2^; a third with unilateral dysplastic kidney and hypoplastic pancreas who developed diabetes during pregnancy^3^; and a fourth who was diagnosed with 17q12 deletion during pregnancy after presenting with bicornuate uterus, early diabetes, hypomagnesemia, fetal finding of unilateral dysplastic kidney, and delivered at 32 weeks with preterm premature rupture of the membranes^4^ (Table 2).

**Table 2:** Outcomes of pregnancies with known maternal *HNF1B* deletion from prior literature

Study	Maternal features (known prior to pregnancy)	Fetal findings	Pregnancy complications	Pregnancy outcome
Morton et al^2^	Diabetes Fatty liver Hypomagnesemia Renal cysts	No anomalies noted	Pregnancy one pre-eclampsia Pregnancy two refractory hypomagnesemia	Term deliveries Macrosomic infants Neonatal genetic testing declined
Morton et al^2^	Cholestasis (outside of pregnancy) Hypomagnesemia	Large fetal kidneys	Diabetes	Term delivery Neonatal hypoglycemia Neonate with 17q12 deletion
Haider et al^3^	Unilateral dysplastic kidney Hypoplastic pancreas	Unknown	Diabetes	Term delivery Child with normal kidneys Genetic testing not reported
Mikuscheva et al^4^	None *HNFIB* deletion diagnosis during pregnancy due to early diabetes	Unilateral dysplastic kidney	Bicornuate uterus Early diabetes Hypomagnesemia Polyhydramnios Premature rupture of membranes	Preterm birth at 32 wk Neonatal intensive care (5 wk) Neonate with 17q12 deletion
Our case	Bicornuate uterus Prior fetal renal anomaly Prior pregnancy with cholestasis and diabetes	None significant in the current pregnancy	Recurrent severe cholestasis Profound hypomagnesemia Diabetes	Iatrogenic preterm birth at 33 wk Neonatal intensive care (5 wk)

Our patient’s combination of clinical findings (including refractory hypomagnesemia, renal cysts, bicornuate uterus, anxiety, diabetes, severe cholestasis during pregnancy, dysmorphism, and family history of a son with congenital renal anomaly and autism) fit the phenotype of the identified 17q12 deletion. 17q12 deletion was the likely etiology of recurrent cholestasis, presenting along with Mullerian anomaly (bicornuate uterus) and a fetal renal malformation previously. In the current pregnancy, the patient had renal dysfunction manifesting as profound hypomagnesemia. Hypomagnesemia likely also affected her prior pregnancies, but was not diagnosed until the current pregnancy.

Although cholestasis and gestational diabetes may be relatively common and even concurrently occurring obstetrical complications, cases with bile acids >100 are uncommon and should prompt a broader differential diagnosis. 17q12 deletion may first present with severe cholestasis during pregnancy and is important to differentiate from classic intrahepatic cholestasis of pregnancy, which is a stand-alone pregnancy-related disorder of multi-factorial etiology resulting from genetic susceptibility (aberrant bile acid transporter/s) and hormonal influence (increased estrogen).^5^ The identification of 17q12 deletion among patients with severe elevations of bile acids during pregnancy may allow for more comprehensive management of this otherwise unrecognized syndrome, and could also distinguish it from progressive familial intrahepatic cholestasis (a group of autosomal recessive disorders with abnormal bile formation that is associated with potentially fatal liver complications).^5^ Knowledge of *HNF1B* deletion or other pathogenic variation in *HNF1B* is important to the counseling and management of affected reproductive-aged women since it is associated with cholestasis (increasing stillbirth risk); worsening of renal dysfunction; and a newborn who may have significant renal disease.^6^ *HNF1B* abnormality should be considered in the differential diagnosis when severely elevated bile acids are encountered during pregnancy, particularly in combination with Mullerian anomaly or renal dysfunction, or fetal renal anomaly.

## Patient reflections

The patient was grateful to have been transferred to a specialty center for multidisciplinary evaluation and management. She was appreciative of the care she received, particularly that despite the need for interventions for electrolyte imbalance and pruritus, her delivery was appropriately timed and went smoothly. She had questioned in the past whether her joint laxity and various other seemingly vague symptoms had been the result of a connective tissue disorder such as Ehlers–Danlos syndrome, but this had never been addressed. She felt that the thorough evaluation she had with genetics during the hospital course was especially insightful, and she was eager to follow-up with pediatric genetics to have further information regarding her children.

## CRediT authorship contribution statement

**Nina Boe:** Writing – review & editing, Writing – original draft, Formal analysis, Data curation, Conceptualization. **Herman Hedriana:** Writing – review & editing, Supervision. **Krishna Singh:** Writing – review & editing, Writing – original draft.
